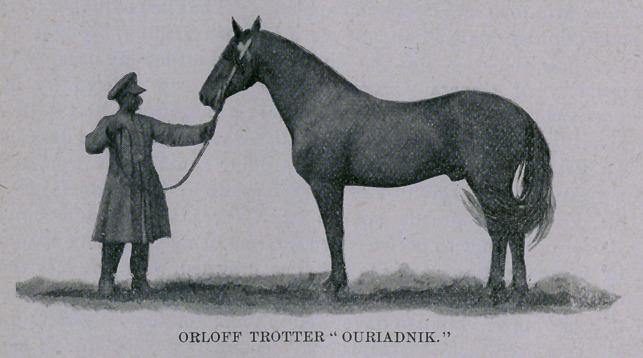# Horse Show—Columbian Exhibition

**Published:** 1893-10

**Authors:** A. H. Godfrey


### THE JOURNAL
OF
### COMPARATIVE MEDICINE AND VETERINARY ARCHIVES.

Vol. XIV. OCTOBER, 1893. No. 4.

### HORSE SHOW, COLUMBIAN EXHIBITION.

By A. H. Godfrey.

At the request of the editors that I should place before their readers my impressions on the Live Stock exhibit at the World's Fair, I submit the following, and trust that it will prove acceptable to at least a few of the lay readers who may occasionally find time to glance over a paragraph or two written in the simple conversational style, as distinguished from the high-class technical matter generally looked for by the learned clientele.

Having been for some years a more or less close observer of the varieties of type which the several breeds of horses present, the first point which struck me forcibly on arrival at the Columbian Exposition was the magnificent opportunity which the Horse Show afforded to make a comparison of the different breeds and to examine the best representatives of each at rest and in action. With this purpose in view I made during the three weeks, commencing August 22, that the show was in progress, daily tours through the stables, held conversations with a majority of the exhibitors with whom I could converse in English, and religiously attended the parade of the several classes before the judges in the arena. I was also enabled, through the courtesy of owners, to take with a small kodak photographs of the leading prize-winners, and I enclose herewith a few prints from film negatives, which I trust will be found of service in illustrating this article.

The Live Stock exhibit consisted of something like two thousand head of cattle and twelve hundred horses and ponies, many of which appeared in the ring in two or more classes, making in all nearly four thousand entries for the authorities to handle. Many of the horses were exhibited by foreign governments and held as priceless, the same being said of hundreds of the prize cattle put into the ring by their American owners. It is therefore extremely difficult to compute the value of the total exhibit, but rating the animals at the low price of, say two hundred dollars a head, we easily run up into the million, and, I think we might with safety put it at twice that sum. As to general excellence I should say that the stock as a whole compared more than favorably with anything of the sort heretofore shown at European exhibitions, and in some sections the exhibit was the grandest and largest that has ever been brought before the public. This opinion is based upon the spontaneous exclamations of competent judges and well-known breeders who came to the Fair merely as visitors, and, I think, prepared to be disappointed and to return home full of adverse criticism.

The provision made for the reception, housing, feeding, exercising and showing of this grand collection of animals was surprising, by reason of the simple style of the buildings and the total absence of anything like friction in bringing the classes before the judges. All live stock trains deposited their loads on platforms immediately adjoining the stables, and animals were led right into their own stalls without coming in contact with occupants of adjacent barns or passing through any building but the one in which they were assigned.

The stabling consisted of about forty frame buildings, many of the larger being L shaped and about 200x40 feet. the smaller barns being of same width but perhaps 100 feet long. All the barns were filled with stock occupying stalls 10x5 feet, or boxes 10x14 feet, and these were ranged down each side, there being a narrow passage between the head of the stalls and the outer walls for storing and distributing feed, and a centre passageway about 12 feet wide throughout the entire length of each building, so that visitors could promenade conveniently and with perfect safety. Every stable was well lighted by windows opposite alternate stalls, and these, with the end doors, afforded excellent ventilation. Over the stalls was a floor where any of the attendants, who cared to do so, could make up their beds and keep their baggage, etc. An abundance of straw, grain and fodder was provided at low rates and delivered daily as required. Water was conveyed through pipes to troughs placed at convenient intervals. The barns were all cleared up and refuse removed in trucks every morning before visitors arrived on the grounds. The barns were marked Section A, B, C, D, etc., to correspond with the sections in the catalogue separating the different breeds. The cattle were of course stabled apart from the horses, and the latter were divided so that it was easy to locate any particular breed.

The Live Stock pavilion, where the judging was done, was an immense building, elliptical in form, some 450 feet long and 280 feet wide, containing galleries capable of accommodating probably 10,000 people, and a show ring that must have been not less than 350 feet in length and 200 feet wide. The surface of the ring was deep tanbark laid on cedar blocks. In the centre of the ring was a covered stand having chairs and tables for the judges and clerks. For the entrance and exit of animals there were wide passageways at the ends and sides of the building, while for visitors there were many stairways at intervals round the outside. Underneath the galleries were club rooms for breeders' societies and offices of newspapers, etc.

The judging continued daily for three weeks, between the hours of 10 A. M. and 6 P. M. The horses were shown at one end of the arena, the cattle parading at the other, the outer edge of the entire circle being generally kept clear for speedy horses to show their paces. $150,000 was awarded in prizes to the competitors by the Exposition Directory, and added to this were several magnificent special prizes offered by the several Live Stock Associations, some of the States defraying expenses for their exhibitors.

The Live Stock exhibit was in charge of the following officials: W. I. Buchanan, Chief Department of Agriculture; Charles F. Mills, Chief Clerk Department of Live Stock; Division A (Cattle), J. B. Dinsmore, Superintendent; Division B (Horses), H. H. Hinds, Marshal of ring; F. J. McMahon, V.S., Chief Veterinarian.

The judges were as follows, except in a few instances where substitutes had to be appointed vice those who were unable to appear, or who were obliged on account of business to leave the grounds before their work was completed:

DIVISION A—CATTLE.

Class 1—Short Horn. J. H. Pickrell, Chicago, Ill.; Consulting Judges—H. C. Duncan, Osborne, Ind.; J. T. Gibson, Deerfield, Ont.

Class 2—Hereford. J. A. Funkhouser, Plattsburg, Mo.

Class 3—Aberdeen-Angus. John G. Imboden, Decatur, Ill.

Class 4—Galloway. David McCrae, Guelph, Ont.

Class 5—Devon. Benj. R. Eldridge, Provo City, Utah.

Class 6—Jersey. Henry V. Alford, Washington, D. C.
Class 7—Holstein-Friesian. Thos. B. Wales, Boston, Mass.
Class 8—Ayrshire. (Did not get the name.)
Class 9—Guernsey. Edward Burnett, Madison, N. J.
Class 11—Red Polled. (Did not get the name.)
Class 12—Polled Durham. D. H. Branson, Altgers, Pa.
Class 13—Dutch Belted. D. H. Branson, Altgers, Pa.
Class 14—Brown Swiss. Wm. Andrew, Lake Forest, Ill.

DIVISION B—HORSES.

Class 23—French Coach. R. B. Ogilvie, Madison, Wis.
Class 24—German Coach. Landstallmeister, Von Oettingen Beberbeck.
Class 25—Cleveland Bay. Alex. Galbraith, Janesville, Wis.
Class 26—Percheron. Thomas Slatterly, Onarga, Ill.
Class 27—Clydesdale. Alex. Weir, Sandelmains, Lanark, Scotland; Consulting Judges—John C. Huston, Blandinsville, Ill.; E. W. Charlton, Duncrief, Ont.
Class 28—Shire. Joseph Watson, Beatrice, Neb.
Class 29—French Draft. Robt. Graham, Claremont, Ont.
Class 30—Belgian. Reiterschafts Director, Von Soldern, Plattenburg.
Class 31—Suffolk Punch. Alex. Galbraith, Janesville, Wis.
Class 32—Hackney. A. J. Cassatt, Philadelphia. Pa.
Class 33—Morgan. R. W. Goodrich, Poultney, Vt.
Class 34—Arab. Rev. F. F. Vidal, Suffolk, England.
Class 35—Americo-Arab. Rev. F. F. Vidal, Suffolk, England.
Class 36—French Trotter. Wm. Bonner, Beaver Dam, Wis.
Class 36½—Russian. M. W. Dunham, Wayne, Ill.
Class 37—Saddle. Charles L. Bailey, Lexington, Ky.
Class 38—Shetland Pony. Eli Elliott, West Liberty, Iowa.
Class 39—Jacks and Jennets. Albert Babb, Springfield, Ill.
Special Class—Mules. Albert Babb, Springfield, Ill.

Leaving the cattle out of the question, and giving attention only to the horses, I remarked that the first week of the show was given up wholly to heavy draft breeds, the second week to carriage horses, and the last week to the lighter and speedier breeds, with the Shetland ponies and the mules closing the show. The exhibitors, it was noticed, were, generally speaking, from the Middle and Western States and Canada, very few animals, if any, belonging to breeders in the East, or residents of England. The Russian Orloffs, Belgians and German Coachers made up the foreign exhibit. A peculiar method of arranging the classes was noticed, the horses five years old or over being all judged together, the four-year-olds together, and so on down to sucklings, the question of height never being considered, and horses lacking size appearing at some disadvantage in consequence. The duties of the veterinarian seemed to be confined to the examination of the horses on entering the grounds and periodical visits to them in their stalls so as to detect disorders or contagious disease. There did not seem to be any system of critical examination of horses for soundness before being turned over to the judges, the veterinarian's advice being simply called for in case the judge did not feel satisfied as to the soundness of an animal in the ring. This resulted in much feeling of hocks, lifting of feet, pressure of throats, and movement of the fingers to try the sight, etc., all of which had a tendency to draw public attention to a horse in a way that there seemed to be little need of. Of course the ring was so large and there were so many animals in the ring at a time that perhaps the visitors on the galleries—and there were often as many as ten thousand seated thereon—could not follow every movement of the judges, and so perhaps examinations which took place may not have caused much trouble. It would have helped matters considerably had the horses been passed upon by the veterinarian before they entered the arena. As it was it appeared, in the heavy classes at least, as though the judges held diplomas and were so keenly alive to all defects that a practitioner's assistance was totally unnecessary.

CLYDESDALES.

The "feathery legs"—Scotch Clydesdales and English Shire horses—were very much *en evidence*. Of the former there were no less than 187 head, and of the latter 50. In the Clydes, owners made very large entries; for instance, 19 head came from Mr. Ogilvie's farm at Madison, Wis.; over 30 were sent by Mr. N. F. Clarke of St. Cloud, Minn.; as large a number by Mr. Robert Holloway of Alexis, Ill.; 15 by Mr. L. B. Goodrich of State Centre, Iowa, and so on. The competition was, of course, keen in the extreme, and the judging by points carried out with such exactness as to leave the experts at the ringside in doubt up to the very last moment, and after the decisions left them wondering "where they were at." In the Clydes we saw heaviness carried to an extreme, as weight in the collar is their principal essential, but there was a grand "look-out" to these horses, a magnificent crest or arch of the neck, and an alertness about them that testified to their determination and will power which destroyed all thought of mere beefiness such as one sometimes gets when looking at that most peculiar of animals the "London dray horse," one of which is as wide as his dray or wagon, and pulls a whole brewery behind him. The toppiness of the Clyde was pleasing, and his line from wither to croup over a magnificently turned posterior quarter was beautiful. The shoulder is more beautiful than would be expected in a draft horse where we look for straightness to some extent, but this is counteracted by the power displayed in the conformation of the hind parts, haunch to buttock and stifle joint, or angle of the ilium, ischium and femur, as the professors say when mystifying us. There is a great deal of finish and style about the Clyde, too, which the winner, Mr. Ogilvie's Macqueen, and the second horse, Mr. Goodrich's world-renowned Macclaskie, showed in a remarkable degree. While the older horses were all imported it was noticed that the majority of the yearlings and two-year-olds were bred in this country, and their condition, shapes and action spoke volumes for their American breeders and gave evidence of their being able to compete with honor in "bonnie Scotland," should occasion arise. It was evident, too, that dealers in Clydes had been careful as to their importations, and it is not too much to say that the Clyde has made for himself a great reputation as a producer of perhaps the grandest type of heavy draught horse that this country or any other can require.

### SHIRES.

The Shire horse, as we see it to-day, is the result of, I may say, centuries of judicious breeding on the part of English farmers and others interested in the strictly agricultural horse. The Flemish stallion put on to the Lincolnshire mare and others of adjacent counties long before Henry VIII lost his temper at the pranks of Anne Boleyn, and a systematic following up of type and perfection of proportion has done wonders, and we see in the Shire a construction well calculated to perform every duty on the farm and make a most economical work horse for the small land owner as well as those who own broad acres. The Shire is extremely deep through the heart, body is roomy, giving space for the "internal machinery," and instead of being "tucked up" he gives one the impression that the "12 quarts" might just as well be served at once as make "two bites at a cherry." The arm and shoulder look as though they were put on for business, not for show, being extremely powerful; the back is short, the line on top straighter than the Clyde, and the posterior quarter longer. Some of the specimens were not "so near the ground" as the last described, but they were extremely neat and clean as to "understandings," with muscles showing distinctly throughout, and not smothered to such an extent with feather. In weight the largest seemed to approach 2,000 pounds, but the majority ranged in the neighborhood of 1,600 or 1,700 I should think, and seemed to be in the very pink of working and saleable condition at that. The action is energetic and easy, and having no redundance of avoirdupois they are handled quickly. Injudicious early importations have caused this horse to suffer somewhat in the market, but they are now coming to the front with a rush, and after what was seen of them at Chicago, I look forward to some interesting details of sales in the near future.

When a horse walks well he is half sold, and the Shire I should say could catch his customer "in a walk." Mr. G. Brown, of Aurora, Ill., of course, won easily with his magnificent Shire stallion Holland Major, than which, I suppose, there cannot possibly be a better. The second horse, Messrs Burgess Bros.', of Wenona, Ill., Light of the West, rather wider than the winner and unusually heavy, took the eye of the experts over the rest in the class. Mr. A. B. Holbert, of Greeley, Iowa, also made a rich display in the Shire Class, but the two first mentioned had the greatest number to put before the judges, and it was a question with them who would come out with the most money. The Burgess Bros. carried off the prize for best collection consisting of three of either sex, also for best four stallions and best four mares, therefore they were "out of sight" at the finish.

### PERCHERONS.

The Percherons, which can safely be considered as the most widely known draught horses in America, came out strong at the World's Fair, and in point of quality were more than equal to expectations. One hundred and fifty were shown. The district of La Perche, in Northern France, has certainly given to the world some splendid specimens of the gray horse, and American dealers seem to have obtained quite a respectable collection of the best sort, if we may judge from what appeared in the Chicago show ring—especially those which were shown by Mr. Dunham, of Oaklawn Farm, Wayne, Ill., who carried off about all the prizes worth having in these classes. Whether having their origin in the Norman or Flemish horse, or horse of Brittany with admixture of

Arabian blood, or that of even the Gascony pony, it must be conceded that the Percheron is a good looker, a well made one, and a horse that sells at a glance. He shows an "upstanding" aspect, is well topped, well rounded, is in capital proportion, and both walks and trots quickly, showing usefulness with a turn of speed as a draft horse not surpassed by any other heavy breed. His feet, too, are said to wear well, therefore of all draught breeds handled the Percheron can certainly not be spoken of as "likely to be returned after a short trial." The French Government have long exercised an influence over this breed, supervising the use of stallions and choosing the mares for perpetuation of type, therefore it is not to be wondered at that a ring of these horses show remarkable likeness to each other and a uniformity of excellence. I hope to include a good picture of a champion Percheron stallion in the collection which accompanies this article, because I can by that means convey some idea of the beauty which eighteen stallions of this breed presented when put into line in the aged class. It was a most impressive sight and a picture which only a Rosa Bonheur could do justice to.

### FRENCH DRAUGHT.

Admitting that France produces many different breeds of horses on the order of the Percheron, but yet distinct from it, I could see no reason for a French Draught Class in the Show, as there was no classification for such fine little horses as the Breton and others from adjacent districts, and it was simply a show of more Percherons, or horses so near to his type as to be taken for him. Mr. Dunham was again successful as an exhibitor, and he really had matters his own way on account of the superiority of his horses.

### SUFFOLK PUNCHES.

Here we saw the "clean-legged" draft horse from the southern counties of England, or to be more exact, indigenous to Suffolk, that has for centuries been noted for straining at a load until it gets down on its knees almost and pulls its heart out. They are a handsome heavy horse, very chunky, low on the leg and with the pulling power well distributed. They are, for the most part, chestnuts with good manes and tails, and this has helped them considerably in the matter of adherence to color, chestnut being notorious for coming through. They can trot and walk too, can Punches, and while, perhaps, the Percheron is their equal in speed —well, well, I was about to say let blood tell, and so I won't say any more, although I cannot omit to praise these English medium-weight draught horses as most excellent generally useful horses for either city, draft or agricultural purposes. As used in England, one horse in the shafts and a leader in chains, tandem fashion, they make a smart, slick-looking team, can move along at a walk with extraordinary loads without appearing to expend much effort, and as they are toppy, well crested, as round as an apple, and possess lots of heart and endurance, it is no wonder they are very extensively used. Mr. Peter Hopley, of Lewis, Iowa, made a magnificent exhibit of these horses, and I understand he is one of the largest, if not the largest, breeder and dealer in them in America. His fine stallion Blazer won all before him, as did also his splendid mare Daffodil. In weight these winners approached 2,000 pounds each, but a figure lower than this would be the average for the breed. Ten stallions and twelve mares appeared in the several age classes, and there was a nice class for two stallions, each shown with three of their get; another interesting class for two mares who appeared with two of their produce, and lastly a collection consisting of two stallions and three mares, all to be four years old or over, this latter filled by Mr. Hopley with a superb lot. The Suffolk Punch is distinctly an old English breed, and while it is said that quite a number of Norman stallions, brought over with or immediately subsequent to the Conqueror, were used in the country lying "just the other side of the chalk cliffs," yet it is difficult to get an Englishman to admit that the Suffolk Punch is descended from anything but the Suffolk Punch. A pretty good reason when you think "it's good enough." It is, however, an indisputable fact that these Suffolk Punches were used generally for agricultural and heavy tournament purposes, and with Norman horses their pictures appear in all the old paintings and prints illustrating the feudal period. The Suffolk blood has worked its way into pretty well all the draft and heavy coach stock in the remote districts, and while judicious selection has bred it out to a great extent, we often hear of the "punchy quarter," the "stocky, punchy sort," etc., etc., in most unlooked-for places. The Suffolk Punch has been well likened to the old-fashioned sort of Saxon man—shortish, thick set, square built, sturdy, with a good many sterling qualities, solid, but lacking somewhat graces and amenities; when moved slow to desist from motion, persevering, of indomitable will, iron resolution and determined obstinacy, not far removed from stubbornness, but honest.

BELGIANS.

The Belgians came next, but as I was unable to see them in the ring, and only had one opportunity to visit their stables, I cannot speak with any degree of assurance regarding them, much as I should like to do so. I therefore pass to the

ARABS.

I give a picture of the champion Arab mare Aga, sired by Njerid (National Arabian); first dam Selica by Gadir of the race Saklavi Djedran (Nejid Hedjaz), premier stallion in the stud Abassie of the Viceroy of Egypt, Abbas Padishah. She is a flea-bitten gray, 15¼ hands high, 10 years old; bred at the Royal Stud at Weil, Wurtemberg; imported to America in 1890 by Mr. Jacob Heyl, of Milwaukee, Wis., the gentleman who exhibited her at the World's Fair. Of all the negatives I obtained at the Exposition, I prize this one the most, only regretting that it is a small "snap-shot" on film, instead of a large 8x10 on glass. There is something magnetic about a pure Arab, and whether it is that we love them because of the "flying steed" stories of our youth, or because the animals are really lovable, I can't pretend to say, but certain it is that no matter what kind of horse we examine, there is generally a preference to anything showing "a bit of the Arab," or "a bit o' blood." I don't wonder that the Soudanese, Bedouins and other natives of the deserts of Arabia strut about like feudal lords and look with disdain upon the Christian, for if they own thousands of such beautiful animals as this mare Aga, they are scarcely to be blamed for preferring the contemplation of beauty and intelligence as contained in their horses to figuring out the intellectual status of a European by gazing at him in passing. As the Chinese consider us in regard to our history and religion, so must the Arab classify us as to our knowledge of horse breeding. All our manufacturing in the horse line will never duplicate the Arab's beauty, although of course we have long ago demonstrated that for practical purposes and a variety of them we can give points to the Sheik in making the nag to fit the shafts or to "stay the distance with the weight up." Stonehenge and hundreds of other eminent authorities have never tired of telling us that it is difficult to obtain an unprejudiced opinion regarding the value of this breed in the stud and its claim to public favor as a useful horse, at the present day, outside of its own country. The Rev. F. F. Vidal, Judge of the Arabs at the World's Fair—a gentleman whom I found a perfect encyclopœdia on this breed, and a most interesting conversationalist—I believe touched the proper chord when he said: "It is for use as foundation stock only that we must uphold the Arab, for of course the structure we have built from it and are now using is so infinitely superior to the old Arabian, that the 'steed' should never be thought of except for freshening up the blood a little now and then." I think those were his exact words, or, at any rate, what he did say conveyed this meaning to me. To describe the perfect Arab is generally as successful as trying to paint a flower which the artist may have seen, so far as giving satisfaction to his audience is concerned. I would much rather trust to the photograph than to any language that I could put together, for, as Mr. Heyl truly says, "in the matter of beauty,

ARAB MARE "AGA."

grace and symmetry the Arab stands unequalled;" it is therefore not for a mere novice to criticise something that has for ages been accepted as perfect. As to prize winning, there was of course no other place but first in her class and the Columbia medal for this grand mare Aga, and I think visitors to the Fair are greatly indebted to Mr. Heyl for placing before them such a magnificent exhibit, and something natural, or I might say a reproduction of foundation stock, to compare with the European "made" article in other classes. Besides this mare, Mr. Heyl showed another aged and a fine two-year-old mare, also a grand young stallion, which made up the Arab exhibit. It was a pity that Mr. Randolph Huntingdon, of Oyster Bay, Long Island, N. Y., did not send on to the Fair his collection of Arabs, as they would have added greatly to the interest of this department, and the contest would have been exciting.

AMERICO–ARABS.

There were fifteen stallions and mares in this class, one of the mares also appearing with her colt and filly for the medal given for "best mare and produce." It was something of a surprise to see these latter put into the ring by Mr. Dunham, of Wayne, Ill., the renowned French-Coach and Percheron breeder, but they made a very nice show, indeed, and he well deserved the medal which he got. In a cross-bred class such as this, one needs pedigrees when studying type, and as very few if any pedigrees were given in the apology for a catalogue, which was on sale at the Fair, I am unable to go into detail on individuals in this section, except, perhaps, those exhibited by Mr. Heyl, whose catalogue I happened to obtain. His yearling bay colt Hassan took first premium in his class and will serve our purpose to some extent. I would like to have obtained the pedigrees of Mr. Hall's (of Toronto) three-year-old first and second stallions Fez and Aldebaran, as then we could have discussed type in horses nearly mature. Mr. Heyl's Hassan colt is sired by Young Belmont, the get of Belmont 64 and out of Ida Wilkes; Belmont being by Abdallah 15 and Ida Wilkes the get of Favorite Wilkes 3257. The dam of Hassan was Hasfoura, a pure bred Arab mare who took third premium at the Fair, and bay with a little white on near fetlocks, and 15¼ hands high, aged. Mr. Heyl tells us that the produce of Arab mares from trotting stallions show the uniformly high finish of the Arabian combined with the action of the trotting horse, and promise to develop into exceedingly fine-looking gentleman's drivers and roadsters. As I understand it, the produce of such a cross would necessarily be "fine;" that is to say, suitable for quick road work in a light rig. As there is quite a deal of blood in the trotter already, and certainly more speed than the Arab can supply, I should think that except for beauty of finish and that delightful Arabian aspect, I do not see why a fresh infusion of "blood" should be necessary. However, I suppose that as a matter of fact the most beautiful specimens of the gentleman's roadster are nearly related to Arab blood on one side or the other, and a bit more cannot do any harm, as it certainly cannot detract from their usefulness in light harness. I take it that it is only the speedy driver and roadster that is meant. The cross t'other way about, that is to say the "Arab on top," I believe has been most successfully tried by Mr. Huntingdon, of Long Island, as his magnificent stallion Abdul Hamid, a brilliant chestnut, delightfully marked and most superbly formed, is something worth traveling thousands of miles to see, and demonstrates the advisability of the cross about as forcibly as anything conceivable. This makes me remark again, the pity of it, that the Oyster Bay Stock Farm did not send any horses to the World's Fair, as especially in this half-bred class for Americo-Arabs would they have been an educator for the visitors. I would not be surprised if this was not also a source of disappointment to the English judge who had expected to be able to get through some critical work in this class and point out the difference between the Americo-Arab and the Anglo-Arab, which latter I believe he himself could have put into the ring had he cared to bring some of his own horses across the Atlantic. In regard to the results of the cross, Mr. Heyl tells us that his object has been to increase the speed qualities of the Arab by its contact with the trotting blood, while retaining the Arabian's high finish. Speed is not to be expected in the first generation, but moderate speed and fine individuality and tractability may be looked for.

### FRENCH COACHERS.

*Fiat justitia ruat cœlum.* Yes, surely, justice must be done the French Coacher, for of a truth no breed at the World's Fair made such a sensation as he did. And he was a surprise too, for I believe I am correct in saying that never before had this breed made his superiority felt so strongly and in such strong competition. Certainly never outside his own country. Responsible for this wonderful condition of things is Mr. M. W. Dunham, the proprietor of Oaklawn Farm, Wayne, Illinois, a breeding establishment which he himself describes as the largest in the world. A pretty tall statement, but I think backed up by solid fact. No less than fifty French Coachers, including foals, represented Oaklawn to prove the ability to put before the public the equal of horses from abroad, and a glance at their extended pedigrees, which abound in details of speed records and historical notes of progenitors, proves beyond doubt that Mr. Dunham has established the most gigantic enterprise in the horse business on a grand foundation, so far as the excellence of the back blood of his stock is concerned; and a moment's examination of the foals put into the ring demonstrates in the strongest manner possible the ability of the breed to reproduce itself, if I may use the term, indefinitely. Size, style, symmetry, action, endurance, and speed are what is claimed for this beautiful, breedy-looking carriage horse, and as the records show their performance of feats of endurance, speed at long distance, and adherence to type generation after generation, it must be admitted that the horse is about all that is claimed for it. I take the liberty, however, of adding, when bred pure. Now, as I understand it, "pure French Coach blood" is something which has only in recent years "been added to the collection," as the showman would say. When we can speak of a breed tracing back for a few centuries, then I think we can with reason talk about pure blood. I believe it is generally understood that the French Coacher is a "made" horse, and "made" what it is in recent years. With the light thrown upon it at the Fair, however, we now understand that we must go back to the time of Louis XIV, when, in order to fill the demand for horses of great elegance, a number of Arabs, Barbs and *stallions from other countries* were imported by the order of the King and bred to the finest mares of

FRENCH COACHER "PERFECTION."

the old saddle breeds of the District of Merlerault and Cotentin, three hundred being concentrated at the Royal Haras of Pin. The produce were called demi-sang (half-blood), a name that is to-day used to designate the French coach horse in France to distinguish them from other races of that country. We are told that it is entirely wrong to suppose that a Percheron mare was used as the dam, as the French have always been opposed to using thoroughbreds upon draft mares. On the other hand the names of imported Arabs appear hundreds of times in the pedigrees of the coach horse engrafted on the mares of the old Cotentin or Merlerault stock.

After the Revolution, and when the roads of France became famous, travel by coach increased and the demand sprung up for stout, stylish carriage horses of the trotting stamp, the govern ment taking the matter up and importing between 1815 and 1833 no less than 1902 stallions, 223 mostly direct from Arabia, best calculated to beget size, endurance, speed and style for carriage work. The French stud book was established in 1833, trotting races were inaugurated, and all trotters under 15¼ hands high were excluded, the distances were two to three miles on a sod track, the horses carrying in the saddle as much as 120 pounds. The weight for four-year-olds is up to 140 and 170 pounds. They maintain that trotting on sod produces high, round knee action, the only truly beautiful movement for a carriage horse; that a high step shortens the stride, and that speed is, therefore, obtained by a quicker movement, the whole tending to a perfect adjustment of stride with rapidity of step, giving the greatest amount of speed with the least expense of power. By picking stallions of most pleas-

FRENCH COACH FILLY.

ing exterior, that have proved their abilities at speed tests, that are large and without coarseness, success is believed to be assured. Two and one-half miles in 7 minutes 42 seconds was the trotting record in 1842, and the best time for five-year-olds in 1891 was 3¾ miles in 9 minutes 44 seconds, many hundreds of horses going a mile in 2.50; no less than 101 horses three years old going a mile under 2 minutes 45 seconds, and 62 horses doing a mile in 2 minutes 40 seconds. In 1887 the French government owned 2460 stallions, 1728 of which were coach bred (demi-sang), and they exported 34,518 horses of all breeds in that year. From the government and other sources there was $1,400,000 given towards the support of their grand trotting races. The Department of Agriculture supervises the Haras or studs, which are in charge of a director-general and inspectors, all of whom are appointed from graduates from the Haras schools. The choicest stallions of different breeds are introduced into different localities and offered for use at nominal fees. These stallions are of three kinds—government stallions, those of private individuals who, after their stallions have passed inspection, are paid by the government from $60 to $600 per annum according to breed and excellence, and other inspected stallions considered good enough for public patronage. The official model, as published in France for the breeders' information and guidance, calls for everything that goes to make an absolutely perfect carriage horse, viz.: size, style, substance without coarseness, action, speed, endurance, beautiful conformation, and freedom from all hereditary unsoundness. Weight is not stated, but height must be 16½ hands. Speed test necessary to the production of a typical carriage horse is put at 4,000 meters in 8 minutes. Now all this is extremely pertinent, and it shows pretty pointedly what is going on in France towards the production of excellence in the way of large carriage horses. The representatives of this breed as seen at the World's Fair were, without doubt, splendid types, and the foals, ten or twelve of which were put into the ring alongside a stallion or two and three or four mares, told a story that there was no getting away from. The pictures of the mare and colt which I have furnished to accompany this article will need no remarks from me to assist them. Probably the picture of the stallion Perfection will reproduce in half-tone better, and I hope it is also used. The stallion Indre, a magnificent upstanding chestnut horse, 16 hands high, 7 years old, winner of gold medal at Paris Exposition, 1889, in a class of 60 stallions open to the world, made a tremendous showing at Chicago, giving us an exhibition of carriage action with a force that I have never seen equaled, and propelling himself with a swing of the hind leg that was simply terrific. A pony at full gallop by his side had all he could do to keep the rein easy, and when it is understood that such a horse could get up extremely high speed in a ring 350 feet long and 200 feet wide, it will be admitted that he must have "brought down the house."

*Ex uno disce omnes* may be all wrong, but records and pedigrees of ancestors tell the same story, and all are authenticated by government officials, the details being published annually. Of course these records are under the pedigrees of the first, second and third generation back, many of the third and all, or nearly all, the fourth sires having been English thoroughbreds, some of them Hackney stallions, etc., etc. The fourth dams are spoken of as "mother of two stallions that trotted in," etc., etc., or "were government stallions," etc. The fact must also not be lost sight of that a great many horses in these pedigrees came to be known by French names, whereas if the matter was hunted out some of them would be found to have been imported under English or other dealers' influence No matter, though, whether it's a "made" horse, or a "pure bred coach horse," it's a grand horse, or, I should say, those I saw at Chicago were grand horses, and if they can sustain their excellence by in-breeding in this country success is assured, but a horse for use in this country must be useful on the mares he meets that are native here, and this is a piece of work which he may or may not be equally successful at. Any country to own such animals as Mr. Dunham put into the ring at the World's Fair is a million dollars the richer by reason of those few individuals, and if they will do all that they appear to be able to do, then untold millions will not count their value. In the matter of prizes, I think I have already said that Oaklawn took the majority, and they made up a silken banner for the parade which held no less than sixty-five first premium and sweepstakes blue ribbons won at the Columbian Exposition in French Coach, Percheron and Arab classes. And now let us get across the channel and talk about the

CLEVELAND BAYS.

The picture of Mr. R. P. Stericker's renowned prize-winner Highcliffe, while not doing the horse justice, will serve to show the difference in type of this breed as compared with the French Coach horse, the one denoting substance and power, the other "extreme breediness." The Cleveland is "long and low," powerful in the forehand, has an "upstanding" aspect, is naturally crested, is remarkably straight on top, running in a direct line to root of dock, the posterior quarter being square, long and beautifully curved down to a well muscled gaskin running to a hock "well let down." The black "list" along the spinal column, and that striking bay color of the body, with points black, are always present in the Cleveland to stamp him as with a sign of his race. 16 hands 1½ inch is the height of Highcliffe, and his weight is 1,480 pounds, at 7 years old. As a breed, it runs from 16 hands to 16 2½ inches, and the weight from 1,200 to 1,450. He is another distinctive English breed, indigenous to one section—this time that district of Yorkshire called the Vale of Cleveland, or Pickering, as the Sterickers, an old Yorkshire horse breeding family, would call it. This Vale has from time immemorial been famous for its carriage horses, hunters, troop horses and hackneys of the highest grade; and it still preserves its character, although the character of the horses themselves has changed somewhat, the use of the thoroughbred having left horses on the hunter type and showy, stylish carriage type rather than the distinct types as enumerated above. The Cleveland Bay, when found pure and tracing into straight Cleveland back blood, is sound, hardy, active and powerful, with great endurance both for draught and for weight, any distance, at a certain speed, under the saddle. The largest and heaviest type have always been favorite coach horses, and the more springy and lightly built were the hunters of days gone by, when the heavy weight farmer and heavier weight hunting squire were not satisfied without six solid hours in the saddle over the roughest ground, ploughed and otherwise, after a fox who, not being compelled to go the pace of to-day, was less skittish about

CLEVELAND BAY "HIGHCLIFFE."

creeping in the open, and did not seek earth until he was completely worn out. The Cleveland has long had the reputation of being the most prepotent coach horse in the world, stamping its own color and form on its progeny with absolute certainty, and its get out of mares of other breeds of about its own stature have always found a market as coach stock. In the matter of speed they have been known to trot a mile in three minutes carrying 196 pounds, and eighteen miles inside of one hour with 250 pounds up, but these are exceptional cases, as is the record of 700 pounds being carried sixty miles in twenty-four hours, four times a week. The most interesting story is that of the Yorkshire merchant who left London on Monday morning, arrived in York, 200 miles distant, on Wednesday afternoon, the same evening went on to Yarm, his home, 40 miles further, which he reached at one in the morning, was called at six A. M., and rode his same old Cleveland mare another nine miles to Darlington, where, when put in the stable, she "ate like a hawk" There were at least fifty of these Cleveland bays exhibited at the World's Fair, and a magnificent appearance they made, eight stallions and three mares being shown by Stericker Bros., of Springfield, Ill.; a dozen by Mr. G. E. Brown, of Aurora, Ill.; the remainder distributed between the Cleveland Bay Horse Company, of Paw Paw, Michigan, Warren & Son, Wis., and others. Mr. Stericker's Highcliffe took first, and Mr. Brown's Eclat ran him very close, the latter having lots of quality, especially in the forehand, the former boasting of best top line, but in the flesh leaning to the heavy side. In the four-year-old class these two exhibitors competed again in the first three horses, Mr. Brown taking first and third, and Mr. Stericker getting in between. Stericker's Dewdrop mare took the cup and sweepstakes, and as she appeared with a suckling colt, she demonstrated forcibly the kind of breeding machine these Clevelands make. As a contrast or foil for the last two breeds described, we will now consider the horses from the land of the Kaiser

GERMAN COACHERS

We ignorant globe-trotters, who have always spoken of the German Coacher as a German Coacher, discovered to our surprise at the Columbian Exposition that he may be either Prussian Trahkenen, Hanoverian, Oldenburg or Holstein, and anything from a medium weight saddle horse to a coach horse or van horse of a decidedly heavy type, in some instances approaching the agricultural horse, though by the pictures presented in the stud catalogues or oil paintings exhibited in the stables of all these breeds, one would imagine that they are all on the order of the French Coacher or the Cleveland Bay. As to the Trahkenen of Prussia, we learn that "it has been bred homogeneous since 1787, combining the best strains of Oriental and English thoroughbred blood grafted upon a select strain of native horses suitable for the purpose, the result after careful selection and judicious breeding being a fixed type of acknowledged excellence, which reproduces itself with great uniformity." Virgil, a grand looking black horse, 16 hands high, six years old, and bred in Walterkehmen, Prussia, belonging to Mr. Jacob Heyl, shows distinctive character and quality; but he, in three removes on top, is into the family of Revolution, Lady Lowther and Filho da Puta, while on his dam's side the English thoroughbred is remarkably prevalent. The information I was able to gain about the character of the "selected mares native to Prussia" amounted to so little that I don't remember whether or not I got any details about them, and there was such a dearth of information respecting the character of horses actually named in the pedigrees, and respecting the nomenclature, and still less about performances of sires, grandsires and great-grandsires, when not English race horses. that we have absolutely no peg handy on which to hang up our enthusiasm Besides all this, there was a lamentable absence of young stock, either full-blooded or half-bred, as an indication of what these horses can get. With regard to Hanoverians, we are informed that for centuries the Prussian Province of Hanover has been renowned for its horses for riding and driving, and that Italy and France draw large supplies from it, and that the boulevards of Paris bristle with horses of Hanoverian origin. It is further claimed that the Hanoverian horse has a noble and regular proportion, high, well set neck, well developed muscles, is strongly built, and has a long step besides being very docile in harness, easy to guide in the saddle and good-natured in the stall.

Now I should say for a "family horse" this description about fills the bill, provided, of course, that the depot is next door; but, I am pleased to be able to say, that this description does not by any means describe the best specimens of the German Coacher seen at the World's Fair. It certainly would not begin to cover Mr. Holbert's Moltke, Amandus, Kaiser Frederic, the mares Lillie and Dora and the Oldenburg Society's champion mare Heldin It would be interesting to study the different types presented in all these German breeds, as the government of the several Provinces named have supervised the breeding of horses, have been distributing inspected stallions among breeding stations, and the German army—a tremendous customer; I suppose the great market for these horses—has for years been drawing its supply from these breeding establishments, as above, if we are to believe all we are told by the exhibitors of the German Coachers at Chicago. The two German judges, Rittershafts Director Von Oettingen, of Beberbeck, and Landstallmeister von Soldern, of Plattenburg, went through their work in the ring in a rather peculiar manner, and gave decisions which decidedly did not appear to be popular nor to meet the views regarding conformation which English-speaking horsemen have invariably been guided by. The men in charge of the horses were rather heavy and heavily burdened in the boots and livery which they wore, and in running their horses on the line seemed to have a predisposition to get under the front feet of their horses and run under their noses, watching their own steps rather than those of the horses, and filling up the time with shouting something which caused the animals to go out of their stride more often than make them improve their speed or style. "Poorly shown" (*Anglice*) expresses it I think, and if these horses are to gain prominence in the American market, swifter runners must be engaged, long lines must be used, and horses must be got into a spirited condition, especially for the show ring, if they are to exhibit what is probably in them. As a good honest pair in a heavy carriage or T cart a couple gave us a show about the grounds. but this pair would not have made much of a surprise in a four-in-hand, either in the wheel or the lead, certainly not the latter. I did not see any of them under saddle. As an array of horses, or a group, they, in consequence of the large number shown, got the attention of the audience, but like the ordinary, common-place actor, when the leading tragedian has left the stage, the parade did not seem to hold the crowd enthralled by any means, and the opinion which I heard on all sides gave me the impression that, taken altogether, the German Coacher was "an honest sort of horse that would find a constant market at a reasonable figure for city general purposes, as distinguished from the quick demand for the other breeds of carriage horses already described."

And now, after taking up so much of your valuable space on foreigners, I will take a dip into my own element, although I shall scarcely go overhead into it, as I am afraid I am tiring you. The spring we now take will deposit us into the

HACKNEYS.

There were thirty-two in this section, and there should have been five hundred and thirty-two, after all the public enthusiasm which the importation of these horses has caused during the last three years, and the vast sums of money which have been laid out to place on this continent the leading representatives of the breed. This large outlay of capital, though, represents for the most part Eastern buyers, and at this Fair there were no horses from the East or from England exhibited in the Hackney classes, a few Canadian gentlemen, like the owner of the champions, Mr. Robert Beith, of Ontario; Mr. Crossley and Mr. Hastings, of same Province; and Western breeders and dealers like Mr. R. P. Stericker, of Springfield, Ill.; Messrs. Thomson & Bland, of Crawfordsville,

Ind.; Burgess Bros., Wenona, Ill.; A. L. Sullivan, Nebraska, and three or four others, comprising the list. As at all previous shows, the horses in this section were of several types and standards, and as the arrangement of the catalogue precluded any attempt to classify animals of a certain height together, but, on the other hand, required exhibitors to show horses of a certain age together without reference to height, there was promised a little difficulty from the start, and, indeed, this objection had been raised in other classes with just as much good reason. The "real old-fashioned type," or at any rate the closest approach to it, was put into the ring by the Canadians, and while their aged stallion, who won in that class, was hardly of this pattern, yet they showed graceful action and a sire in their six-year-old Jubilee Chief; a particularly sweet and fetching winner in the three-year-old chestnut colt Ottawa, son of Lord Derwent (1034); took third prize with a well-topped two-year-old colt, Star of Mepal II; won championship with the aged stallion already mentioned; got second prize in the aged mare class with Lady Coking, probably the best "real old-fashioned one at the Fair"; picked up first prize with their four-year-old mare Lady Bird; gathered in first and second with their three-year-old mares Winnifred by Wildfire (Bonfire's sire) and Lady Aberdeen by Lord Derwent, and therefore half sister to the colt Ottawa, mentioned above, and to conclude, carried off the mare championship with the leading three-year-old Winnifred. Mr. Robert Beith and Mr. Crossley deserved all these prizes without a shadow of doubt, as they had a superabundance of all the good points in their individuals as compared with the points shown by competitors in their classes; but how much more interesting and exciting would it have been had the host of horses of similar excellence which the East can show been paraded in the Columbian arena. It was noticed that typical representatives did not always exhibit the most quality or the most action, they evidently lacking the latter on account of poor handling or training. Action was often the only thing to depend upon, with quality and manners to help out. It was also noticed that while some of the Western gentlemen had evidently dealt with horses of extremely useful make up as offerings to farmers and others interested in the development of the larger harness horse, it was quite apparent that a few of the exhibitors had been influenced in their purchases in Hackney districts of England by the saleable qualities of their horses in the public market, rather than by their adherence to high-class type, such as is depended upon in England to reproduce true-shaped representatives of the old-fashioned Hackney breed. The exhibitors, however, showed rare pluck when they brought their horses so many hundreds of miles to the Fair, fully expecting to meet and be beaten by the Eastern high-priced animals that are descended from prize-winning ancestry, and which have been kept in constant show condition without regard to expense. That these latter did not appear is to be regretted of course, from a public point of view, but it should be understood that the great social event which takes place in November at Madison Square Garden, New York, is a Hackney Waterloo not to be played with, and when it is further understood that horses in course of preparation for this great event have also to be shown at such fairs as White Plains, Danbury, Rochester, Boston, Philadelphia, and other places in the Eastern States, some allowance should be made for the failure of their owners to appear in competition at Chicago, which, although the centre of observation for the whole world just now, is far removed from the Eastern base of stud operations, etc., and quite an expensive point to reach, as well as a place where horses with contracts to fill would be compelled to stay for not less than three weeks at a time, when three weeks is something too valuable to lose for the sake of publicity or the small prize money offered by the World's Fair.

In this connection, speaking of prize-money, I think it was rather an invidious distinction to rank the Hackneys, Suffolk Punches, Belgians, Morgans, Kentucky and Missouri Saddle Horses, Jacks and Jennets at $110 for highest class prize against $150 for other breeds. It was also very unjust to put the Shetland Ponies and Mules at $100. A World's Fair should not discriminate among breeds. The Mule is just as much the result of careful breeding and judicious mating of animals as the grade Clyde and Shire, and the Shetland Pony is just as much a necessity, if only for children's use, as the French Coach or Cleveland Bay. The Jack has certainly proved a godsend to the railway contractor and agriculturist of this country by reason of the Mules he has begotten, fit for a use under circumstances where the horse would be absolutely of no value. But to come back to the Hackneys of Yorkshire, the Midland Counties and Norfolk, as shown by representatives of families indigenous to these sections of England, which were put into the Columbian ring, we have added little to our store of information on type, and are only more firmly fixed in our opinion as to the manner in which this country has been supplied with indifferent specimens, many of them not calculated to reproduce any particular type, or even transmit any particular points of excellence to whatever progeny they may get out of native mares. It is this departure from fine individuality typical of a race on the part of importers, whose only object is to sell a horse or two at a small profit, having purchased it originally at a low price, that floods a country with "ordinary" horses, who having no character of their own are unable to transmit any, and soon become swallowed up in the mass of stuff of their own stamp that they meet in a place like the United States. If European countries, who have always had more or less good foundation stock of one kind or another to breed to, have considered it necessary to place the matter of horse-breeding under the supervision of trained experts, and have found it advantageous to set aside annually large sums of money for the encouragement of speed competitions, and to pay private parties for the use of their stock horses, does it not appear that similar arrangements for the improvement of horse-breeding as a national enterprise, and for the supervision of importations, could be adopted in this country without fear of injury to the industry as carried on by individuals or stock companies?

Breeding societies and the publishing of authenticated stud books are doing much to this end, but their work is only proceeding gradually, and is by no means able at the present time to cope with the difficulties that threaten to overwhelm the little good that the societies are able to accomplish. Before closing I suppose I am expected to describe the several types of Hackney as exhibited in the ring, how they differed from each other and in what manner they approached or departed from the "old-fashioned original type." To do this to my own satisfaction would take up more space than I could expect you to reserve for my exclusive use, and in order to demonstrate plainly what I meant it would, I think, be necessary to publish diagrams, or in fact photographs of living horses well known in the show ring, bred on different lines. I therefore will not bore you with any further remarks anent the Hackney, at least not at this writing, except in so far as to state that in height the true pattern ranges from 14 hands or 14.2 to 15.2, is able to move the scale at 850 to 1,200 pounds, in a few instances 1,300, has an outlook almost as "breedy" as the picture of the French coach, but nearer that of the Cleveland Bay portrait which I give; is wide chested and strong in the shoulder without coarseness; has shoulder set sloping—a point of beauty much dwelt on; good girth for his size; wither clean and rather high, and at the bottom of a beautifully curved line which forms the crest running up to the poll; a small ear, alert and well pointed; forehead broad and running down to not exactly a bony, but nevertheless a clean face, which has in all the best specimens a peculiar but pleasant "nag-like" formation, which must be seen in life and cannot be described; the back short, wide and straight, denoting strength to bear weight and as a point of beauty; a wide loin that will bear pressure, and is double, showing to some extent the gutter between muscles; the croup well united to the loin, well muscled, and running almost, but not quite straight back to the dock, which latter should be strongly set in and high, leaving the proper curved line around the buttock and down to gaskin to constitute the beauty of the "real nag quarter." All legs should be short, sturdy, well muscled, tendons well separated, bone big and flat, knees wide in front, hocks clean and strong, pasterns rather short, strong and slightly oblique; feet round and perfect in every particular, it being most important in the legs of a Hackney that the arms are inclined to length, but below the knee and hock short bone, and the hocks very close to the ground. Action should be high, arm to be lifted with a snap, sending out the knee and then the foot with brilliancy and finishing the stride some distance ahead by lightly touching the ground, all this taking place in true time, while the hind legs have been sent well under the body with a high flexion of the hocks so as to uphold the weight or balance the whole fantastically to some extent, and insure perfect rhythm, or, as we say, the 1, 2, 3, 4 step that is so delightful to drive in a two-wheeled perfectly balanced vehicle on a well kept road, the "nag" arching his neck naturally, so as not to pull on the bit, which latter should be adjusted in such a manner as to permit of one hand doing all the work by a light touch of the finger, and a word or a chirrup, or a fairy stroke of the whip on the flank or loins keeping the horse keyed up the entire duration of the drive. This to the lover of a prize-winning and really useful Hackney is heavenly, and this is what all the demonstrations on the tan bark are meant to convey to the uninitiated.

### MORGANS.

Had there been no other attraction but Morgan horses billed for the World's Fair, I would have attended the show with just as much eagerness as I did, for I had promised myself a rare treat in examining at close range representatives of the world-renowned Vermont Morgan breed. I expected to see hundreds of representatives of the much lauded type put into the ring, and I was fully prepared to see mares that would have nicked well with the Hackney to produce a perfect driving cob of sweet combination and as hard as nails. I was disappointed. Of the old type, as I had been led to understand it, I saw some 13.2 to 14 hand ponies, delightfully formed, with the sweetest of heads and necks, the most beautifully turned bodies, and with flowing manes and tails—one or two had wavy tails, I noticed, and hard, but not the best formed legs. They were ponies though, and ponies are not my specialty. The mare who took the championship and first prize in her class, Mr. Battell's Jessie, was particularly rich in quality, and for this, if for no other reason, she well deserved a prize. As old-fashioned type (that which has been pointed out to me for three or four years, and which I have persistently looked for) was not recognized in placing the champions at the World's Fair, but rather what seemed to me to be speedy conformation combined with slightly more size than the old sort showed, I conclude that it was the horses best calculated to trot quickly or to beget trotters that would sell well, which were deemed the best representatives of the Morgan breed.

Then I suppose we must in future accept the Morgan type as merely a "speed type." If so, then Morgans are trotters and should be relegated to the trotting class, in which no particular type is looked for or bred for. Are we to say "Good-bye Morgan"? It is, then, to be business versus sentiment hereafter, and simply breeding useful and saleable horses, without adherence to any particular type, notwithstanding that the lesson which the world acknowledges it has learned is that without type horse-breeding nations have never yet been able to retain uniformity or prepotency that is to be depended upon to reproduce characteristics most highly esteemed in individuals—except, of course, that one point, which may or may not have been satisfactorily settled, viz., speed at the trot. Is it because the old Morgan has degenerated, or has dwindled down to pony form and is in consequence no longer profitable, that this new ground has been struck? Or do the signs of the times read "Speed and size"? Or has the Morgan always been a pony which the old people were content with, but which the new regime (educated as it is by the influx of size and style as instanced in the French Coach, Cleveland and Hackney, which are catching the market rapidly) consider only useful in out-of-the-way country places such as Justin Morgan might have held school at? Shade of Linsley! I wonder who will keep his resting place green now. And yet he only had a 14 hand horse that weighed about 950 pounds to build his Morgan structure on. Then again, the bright chestnut son of this Justin, which was known as Sherman, was only 13¾ hands high, weighing 925 pounds. Granted that Sherman's son Black Hawk went up to nearly 15 hands and weighed about 1,000, but Mr. Linsley told us that Black Hawk's dam was described as a black mare, half-blood English, a very fine animal and fast trotter. Black Hawk's son Ethan Allen, out of a medium sized mare of Messenger blood, was 15 hands and weighed just 1,000 pounds, and his pictures show him to have been a beauty. The English, or some blood very like it, therefore, had got in some work by this time through the dams, and as this later infusion gave size, beauty and speed, it has always been an enigma to me why the small originals on the sire's side are so religiously referred to. And it will be more curious hereafter, if the World's Fair type is to be held up as the proper thing, because on the banner of the new crusade the watchword is "Speed and size," and underneath "Old type Morgan" crossed out. This was the identical banner which seems to have been carried by Col. Jaques of Ten Hills Farm, Charlestown, Mass., who owned or used Sherman in 1831, and who evidently also owned him in 1832 when the horse stood at Dover and Durham, N. H., where he must have served the mare who afterwards dropped Black Hawk. Now Black Hawk had size and style, which was what the Colonel was after. I never like to insinuate, but is it not strange that this same Col. Jaques, of Charlestown, Mass., a gentleman known as a great breeder of horses and other animals, also owned the English Hackney stallion Bellfounder, who came to Boston in 1822 and stood him in and around that city and later in New York State, getting beautiful horses of some size, finish and style out of "half-bred English mares" and others. Bellfounder was a fifteen-hand horse of undoubted Hackney pedigree, and came of trotting lineage way back to a point in the history of Norfolk, England, beyond which it is unnecessary to go. Well, no matter whether the original Morgan type was good, bad or indifferent, and whether or not it was improved by contact with English blood, first principles now seem to obtain, and we must admit that the winners, when not discussed in regard to type, are beautiful horses, very sweet, breedy, and probably able to show a rare turn of speed.

### KENTUCKY AND MISSOURI SADDLE HORSES.

Truly a magnificent exhibit and one that proved conclusively that saddle horses with the several peculiar gaits, such as the running walk, fox trot, rack, etc., in addition to the walk, trot and canter of what is known in the East as the perfect park hack, is a delightful horse under the saddle, and able to save himself by the extra gaits without allowing them to interfere in the least with the regulation park hack motion. General Castleman, of Louisville, Ky., a gentleman of most pleasing finish and address, showed his superb mare Emily in a wonderfully scientific manner, getting out of her all the gaits mentioned, as well as many of the movements of high-school, which latter, however, are not deemed essential or necessary by the General in a good saddle horse. While this mare Emily was a wonder, there were several horses approaching her standard, ridden and dexterously shown by the General's sons, and after winning several first prizes in large classes, the National

"MONTE CRISTO, JR."—KENTUCKY SADDLE HORSE.

Saddle Horse Breeders' Association presented to the General their first silver cup as a memento of his success at the World's Fair. The picture which I send you for this department is that of Monte Cristo, Jr., a magnificent specimen of the Kentucky saddle horse, and as stately a bit of horseflesh as ever entered a ring. Mr. Crenshaw, of Louisville, Ky., was the owner of this splendid animal, and showed him in all the paces exactly and with almost the same degree of cleverness as General Castleman had used. The means adopted by these riders in manœuvering their horses were not discernible, as all was effected by a slight pressure of the knees or thighs, and an imperceptible pressure of the fingers on the reins. The horses were bitted with curb and snaffle, English style, and the dress of the rider consisted of felt hat, sack coat, and either the trousers with side stripe, as for the park, or breeches and jack boots. There was none of that pulling of manes and ears and signing with the hands waving in the air, as was sometimes resorted to by riders from Missouri. These latter, though, put a most magnificent array of horseflesh into the ring, showing finished style, all the gaits, great speed, tractability and thorough proficiency as saddle horses.

One of the most attractive features of the show was the competition among these saddle-horse exhibitors for the championship for aged stallions. After a most exciting contest, in which speed, endurance, manners and ability at all the gaits on the part of the horse, and dexterity and cleverness on the part of the rider, were severely tried, Mr. J. Bridgeford, a gentleman who must have been about seventy years of age, was declared the winner, and as the result was declared, the house rose and saluted the veteran rider with loud and continued applause. The class calling for best collection of one stallion, one mare and one gelding, brought five sets into the ring, and the cup presented ultimately became the property of General Castleman. Another cup, offered by Mr. Buchanan, Chief of Department of Live Stock, for the best stallion, mare or gelding, was won by Mr. Crenshaw's handsome dark bay Monte Cristo, Jr., and to finish up a riding contest was won, after a most brilliant exhibition of horsemanship, by Mr. Bridgeford, the old gentleman already spoken of. Never were saddle horses shown better nor to better effect, the ring being admirably adapted to such exhibitions, and the galleries crowded with not less than ten thousand people every time the horses appeared. The owners of these horses claim to have bred in them for several generations all the gaits which they show, and declare that they are all more or less present in the young foal, needing only direction and dexterous handling of the youngsters to be developed up to the correct pitch for a gentleman's service. The horses are all beautiful, breedy-looking animals, some rather long, but the majority not so, and on the other hand, as a whole, exceedingly well turned, although not more than half of them approaching the conformation of Monte Cristo, Jr.

### RUSSIAN ORLOFFS.

If not the most attractive feature of the whole horse exhibit, at least these were very near it, and as far as their showing in their stalls certainly a long way ahead of anything else on the grounds. Their stable was a parlor rather than a barn. There were eighteen of the Russian horses, light, heavy and draught, in one stall 150

feet long by about 45 feet wide. Every horse stood in a box 10x12 feet, side on to the visitors. The entire front of each box was removed, and the back and sides of the box lined in red bunting (for a white horse) and in blue bunting (for a black horse), which had a tendency to make each animal show off better. Half way across the box a bar covered in red bunting was stretched, the horse behind it standing on clean deep straw, the visitor, if he wished, standing on a mat on the front half of the box floor. Everything was carried out as if on dress parade, the grooms all in Cossack soldier's uniform. Over the head of each stall was a richly illustrated badge setting forth the owner and name, etc., of the horse. Every evening about five o'clock these Russian horses were taken from their stalls, paraded through the grounds and marched off to their sleeping barns at some distance outside the

ORLOFF TROTTER "OURIADNIK."

grounds. Every morning they were all paraded back into the show stalls. All this I should say ought to be useful to those interested in showing off stock at horse shows to the best advantage. These Russian horses were, generally speaking, of four kinds: Light and Heavy Orloff Trotters; Orloff Saddle Horse; Russian-Arab and Orloff-Arab; and Light Russian Draft. The Heavy Orloff Trotter, while being speedy and full of endurance, was not over fine, in fact rather coarse; but standing or in action they show up grandly, the stallion Ouriadnik, whose picture I have given, being fully 16.2 hands, but I should say nearing his 15th year. In action he has a very springy gait, and can go very fast, taking a very long stride without the extraordinary swing of the American trotter behind, but with a rounding action in front that would lose him a race here below the 30 mark, I think. He is a very handsome horse, however, and I should suppose one of the leading representatives of the Orloff trotting family. He is a black horse with gray hairs. This horse was sent to Senator Stanford, of California, by the Russian Government. For a really speedy one Count Dimitry's dapple-gray Tchistiak, five years old and nearly 15 hands, I think was best, but he was nothing very attractive in point of finish or quality. An old white horse called Oussan, approaching 17 hands high, was superbly formed, with clean head, neck well formed, aspect good throughout, straight on top, not too long where most of the Russian horses are, although over the quarter he was long enough, which was saved by the position of his dock. The Saddle Horse Priyatel, black, nearly 17 hands high, and about five years old, was a wonderfully made one and as beautiful as anything at the show in my humble opinion. He was a good walker and at the trot went clear and straight. I ought to have given you a picture of this horse, as I believe I took one when I got Ouriadnik's portrait

Several of these Russian Orloff horses, most of them, by the way, stallions I believe, have been sold by Captain Ismailoff, who conducted the Russian Orloff exhibit at the World's Fair. Mr. John A. Logan, Jr., I understand, has purchased the Saddle horse, the draft stallions, and a chestnut called Beckboolat; while Mr. E. D. Stokes, of the Hoffman House, has taken four of the trotters including Tchistiak. I believe it was the original intention of Captain Ismailoff to travel the Russian exhibit on tour through the country, but as these sales will deplete his stock, I suppose he will simply exhibit what he has left at the New York show before he takes them back to Russia.

With the exception of the Shetland Ponies and the Jacks and Jennets and the Mules, I believe I have now spoken on every breed exhibited, and with your permission I will leave these until some future time, as the Shetlands require mention in detail in order to do justice to the large number of exhibits made by Mr. Hawley, of Pittsford, N. Y.; Mr. Hoag, of Maqoeketa, Iowa; Mr. Watkins, of Detroit, Mich.; and Messrs. Robert and James Lilburn, of Emerald Grove, Wis., the last of whom had the smallest pony—I was going to say in the world—called Toy, 27 inches high, 3 years old, and weighing just 100 pounds.

Before closing, allow me to add my tribute to the many which must have already appeared in the press respecting the kindness and courtesy extended to every exhibitor and others interested in the Live Stock exhibit, by Mr. Buchanan, the Chief of Department, and his secretary, Mr. Mills, both of whom were indefatigable in their efforts to arrange for the personal comfort of all who had in any way business connected with the exhibits, and who would have found great difficulty in obtaining proper and reliable information and permission to enter the arena, etc., had not these officials gone out of their way to assist them.

## Figures and Tables

**Figure f1:**
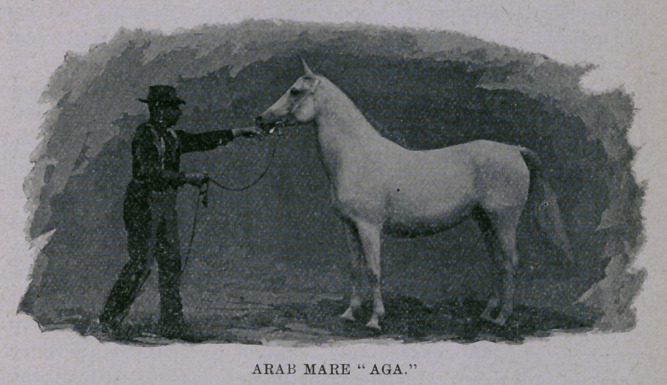


**Figure f2:**
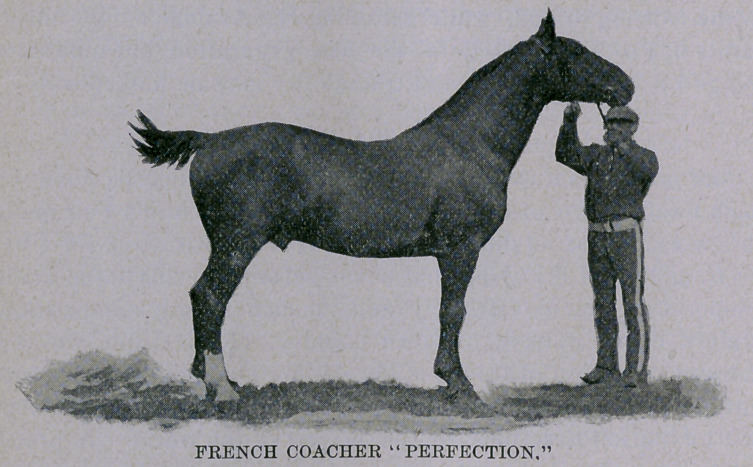


**Figure f3:**
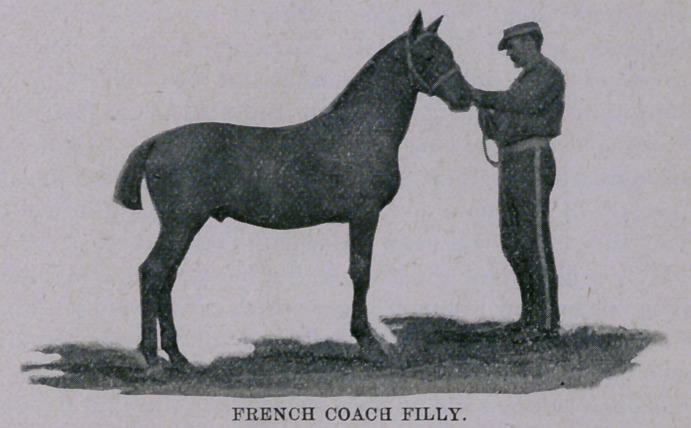


**Figure f4:**
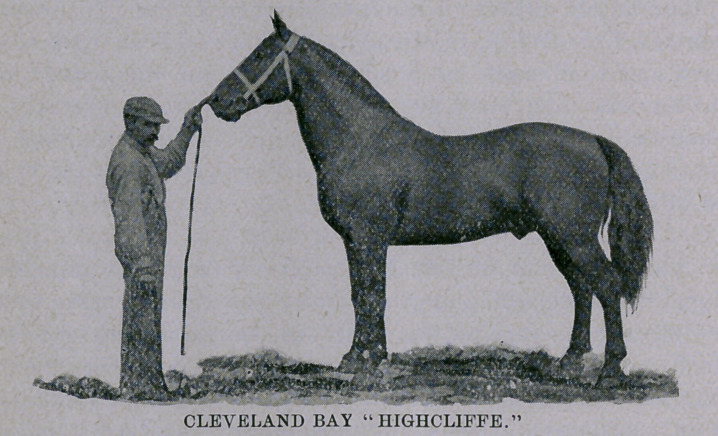


**Figure f5:**
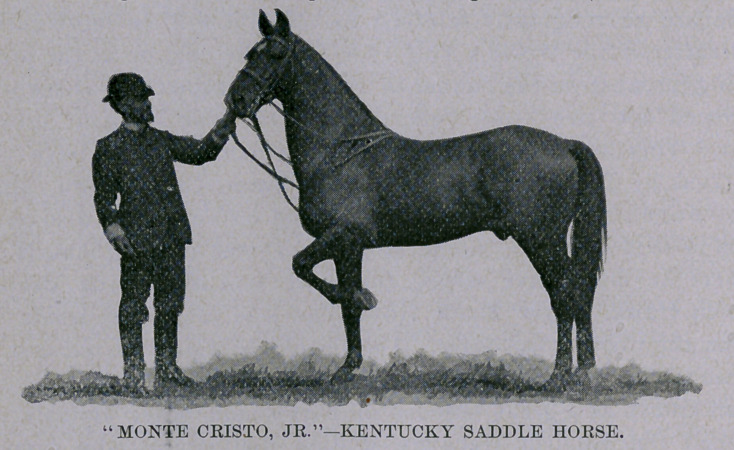


**Figure f6:**